# GAL Regulon in the Yeast *S. cerevisiae* is Highly Evolvable *via* Acquisition in the Coding Regions of the Regulatory Elements of the Network

**DOI:** 10.3389/fmolb.2022.801011

**Published:** 2022-03-14

**Authors:** Rajeshkannan, Anjali Mahilkar, Supreet Saini

**Affiliations:** Department of Chemical Engineering, Indian Institute of Technology Bombay, Mumbai, India

**Keywords:** *S. cerevisiae*, galactose, GAL regulon, gene expression kinetics, evolvability

## Abstract

GAL network in the yeast *S. cerevisiae* is one of the most well-characterized regulatory network. Expression of GAL genes is contingent on exposure to galactose, and an appropriate combination of the alleles of the regulatory genes GAL3, GAL1, GAL80, and GAL4. The presence of multiple regulators in the GAL network makes it unique, as compared to the many sugar utilization networks studied in bacteria. For example, utilization of lactose is controlled by a single regulator LacI, in *E. coli*’s lac operon. Moreover, recent work has demonstrated that multiple alleles of these regulatory proteins are present in yeast isolated from ecological niches. In this work, we develop a mathematical model, and demonstrate via deterministic and stochastic runs of the model, that behavior/gene expression patterns of the cells (at a population level, and at a single-cell resolution) can be modulated by altering the binding affinities between the regulatory proteins. This adaptability is likely the key to explaining the multiple GAL regulatory alleles discovered in ecological isolates in recent years.

## Introduction

Genetic circuits are evolvable. Depending on the precise environmental niches, acquisition of a mutation could alter gene expression dynamics more suited for survival and growth. The changes in the network dynamics could be facilitated by two types of mutations. First, mutations which change processes like transcription and translation, and hence, shape regulatory networks (Wray, 2007; Kim and Przytycka, 2012; Hill et al., 2021). These mutations change the timing and levels of transcription and translation. On the other hand, mutations could also change protein activity, and as a result the affinity of a protein with DNA or another protein; resulting in downstream changes in gene expression (Golding and Dean, 1998). While several examples of the first kind are known, relatively fewer examples of changes in expression patterns by protein modifications are known (Lewontin, 2002; Rodriguez-Trelles et al., 2003).

**FIGURE 1:**
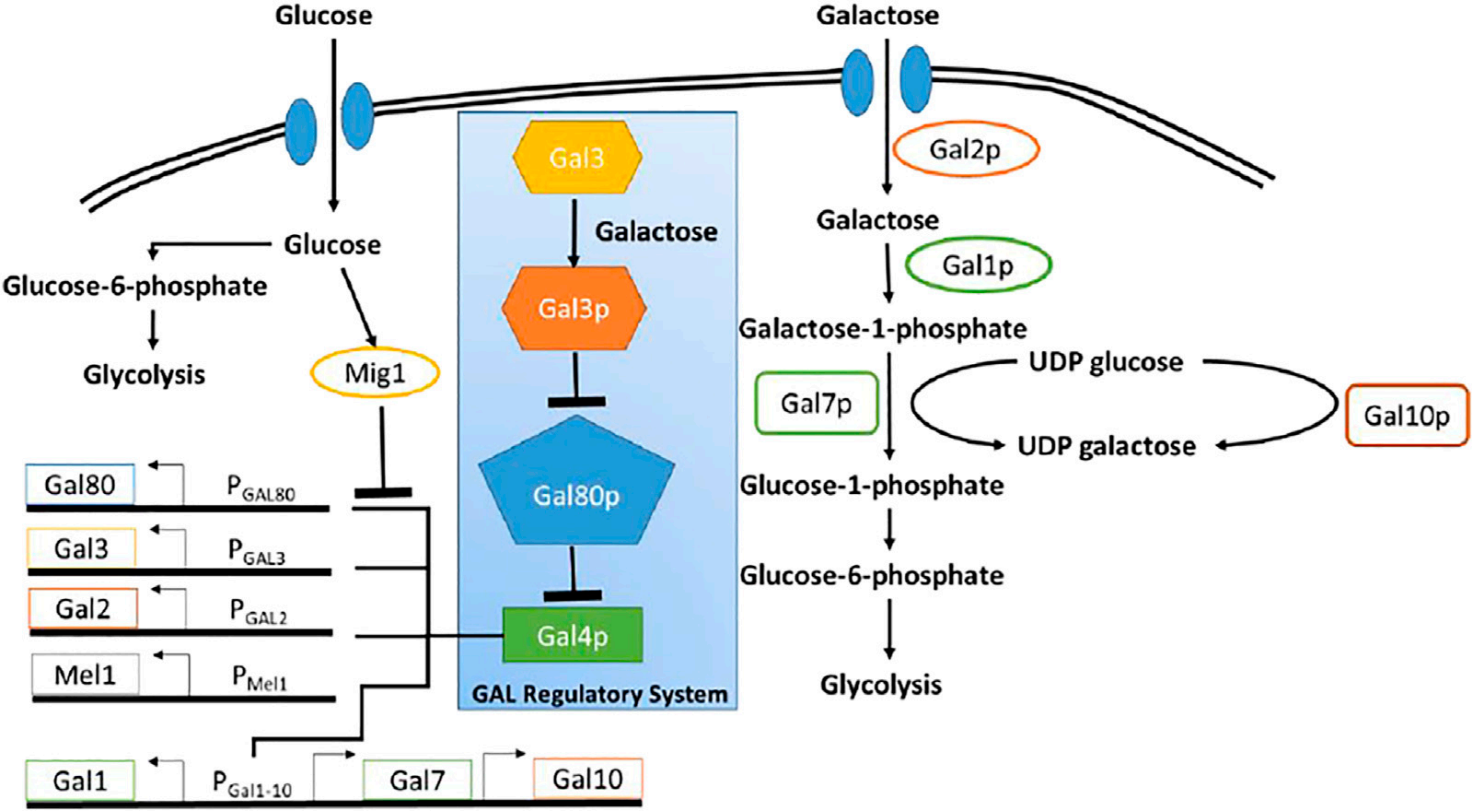
Schematic of galactose and glucose metabolic pathway in Yeast. Blue box highlighted the regulatory network pathway of GAL genes. Glucose as a primary carbon resource, suppress the GAL genes through Mig1 protein. In the presence of galactose and in the absence of glucose, GAL4 protein activates the GAL genes.

The GAL regulon in the yeast *Saccharomyces cerevisiae* (*S. cerevisiae*), which enables the organism to utilize and grow on galactose, is one of the most well-studied regulatory networks in yeast (Bhat and Murthy, 2001). The regulatory network, is briefly explained below (Figure 1). All genes involved in utilization of galactose are under the control of a transcriptional regulator, GAL4p. Gal4p binds to and drive expression from several promoters, which control expression of the GAL regulon (Chasman and Kornberg, 1990). In the absence of galactose, Gal80p binds Gal4p, leading to formation of a Gal80p-Gal4p protein complex. This sequestration of Gal4p thus switches OFF expression from promoters of the GAL regulon. In the presence of galactose, however, the signal transducer Gal3p, binds galactose and in its activated form (Gal3p*) binds Gal80p, forming the complex Gal3p*-Gal80p. The Gal3p*-dependent sequestration of Gal80p, thus frees, Gal4p to activate gene expression from the GAL regulon promoters (Bram et al., 1986; Johnston, 1987). Galactose is brought into the cell via the galactose-specific transporter, Gal2p (Chiang et al., 1996; Hawkins and Smolke, 2006). Upon entry, galactose is first activated upon by the galactokinase, Gal1p (Zenke et al., 1996).

Laboratory work in the 1970s led to isolation of several alleles of GAL regulatory proteins (Douglas and Hawthorne, 1966; Nogi and Fukasawa, 1984; Salmeron et al., 1990). More recently, analysis of yeast isolated from ecological niches has revealed that several alleles of the regulators in the GAL system are present in nature (Wang et al., 2015; Lee et al., 2017). Analysis of several thousands of these isolates was used to reveal that distribution of these alleles is non-random; and certain combinations of alleles confer a greater fitness than others (Boocock et al., 2021).

In this study, we develop a quantitative model to study gene expression dynamics in the GAL system. Several existing models to study this system exist. However, we use the model to ask the following question: how does changing the allelic combinations make to the dynamics of gene expression of the GAL network? Are there specific regulatory elements in the GAL network which control the system’s sensitivity, evolvability than others? We particularly focus on the Gal4p-Gal80p and Gal3*-Gal80p interaction. Towards this, we first develop a quantitative model, and study the system’s behavior at a population as well as at a single-cell resolution. We then validate our model through analysis of the wild type laboratory strain *S. cerevisiae*, and two distinct regulatory mutants. Thereafter, we analyze our model for studying the regulatory evolvability of the network. Our analysis reveals that in the regulatory structure of the GAL network, the gene expression behavior is highly evolvable via acquisition of mutations among the several regulatory elements in the network, thus, suggesting that the GAL network is highly evolvable, and suggesting an explanation regarding the high allelic diversity among its regulatory elements in yeast isolated from ecological isolates.

## Materials and Methods

### Mathematical Modeling of the GAL Network

In *S. cerevisiae,* enzymes encoded in the GAL regulon control metabolism of galactose. Seven proteins play a role in the metabolism of galactose. Among them, there are three enzymes involved in the catalytic pathway to convert galactose to glucose-6-phosphate and there are four other enzymes involved in transportation of galactose and activation of GAL genes (Sellick et al., 2008). Figure 1 shows the schematic diagram of the utilization of glucose and galactose to produce biomass through glycolysis process.

Both glucose and galactose have specific transporter through which those sugars are imported into the cell. Glucose is transported by HXT proteins and galactose is imported through Gal2p. Glucose represses the metabolism of other sugar through global carbon catabolic repression. Therefore, once glucose entered into the cell, the galactose gene network shuts off because of the repression by glucose molecules. Once glucose is completely utilized, the repression is relieved and the galactose gene regulatory network resumes the production of GAL genes. The GAL genes include GAL1, GAL2, GAL3, GAL80, GAL7, and GAL10.

Gal1p, Gal7p, Gal10p constitute the enzymatic pathway (Leloir pathway) involved in the process of converting galactose into glucose-1-phosphate (Sellick et al., 2008). The Gal1p and Gal3p proteins become active in their signal transduction role only when they bind galactose. While Gal3p is exclusively a signal transducer, Gal1p is a galactokinase but also retains some signal transduction activity (Lohr et al., 1995; Platt et al., 2000).

### Modeling of GAL Regulatory System

The modeling of GAL regulatory network based on the deterministic approach adopted in (Venturelli et al., 2012). The rate equations for all the GAL proteins are given below for a single cell. We assume that the environment is a chemostat, and that the extracellular concentration of sugars is constant with time. The rate equation for GAL1 protein is given as follow,

$$\frac{d\left[G1\right]}{dt}=\alpha_{0G1}+\alpha_{G1}\left(\frac{{\left[G4\right]}^{n1}}{{\left[G4\right]}^{n1}+K_{G1}^{n1}}\right)\left(\frac{K_{R1}^{nG1}}{{\left[R\right]}^{nG1}+K_{R1}^{nG1}}\right)-k_{f1}\left[G1\right]\left[B_{i}\right]+k_{r1}\left[G1*\right]-\gamma_{G1}\left[G1\right]$$ (1)

The above equation contains the production rate of Gal1p through basal expression and induced expression through transcription factor Gal4p, which has multiple binding sites and the binding mechanism is modeled as Hill function with coefficient, *n1* equal to 2. The glucose repression is also modeled using the Hill equation for repressor with Hill coefficient, *nG1* equal to 1. The third and fourth terms describe the activation of Gal1p protein when binding with galactose. The last term in the equation describes the degradation of Gal1p.

The rate equation of Gal2p is as follows,

$$\frac{d\left[G2\right]}{dt}=\alpha_{0G2}+\alpha_{G2}\left(\frac{{\left[G4\right]}^{n2}}{{\left[G4\right]}^{n2}+K_{G2}^{n2}}\right)-\gamma_{G2}\left[G2\right]$$ (2)

The rate equation of Gal3p consists of production, transformation, and degradation term. The production terms depend on the basal rate and induced expression rate with glucose repression. The active form of Gal3p, Gal3p* (represented as G3* in the equation) forms when Gal3p binds with galactose. The induction and the repression rates are modeled using the Hill equation with coefficients *n3* equal to 2, and *nG3* equal to 1.

$$\frac{d\left[G3\right]}{dt}=\alpha_{0G3}+\alpha_{G3}\left(\frac{{\left[G4\right]}^{n3}}{{\left[G4\right]}^{n3}+K_{G3}^{n3}}\right)\left(\frac{K_{R3}^{nG3}}{{\left[R\right]}^{nG3}+K_{R3}^{nG3}}\right)-k_{f3}\left[G3\right]\left[B_{i}\right]+k_{r3}\left[G3*\right]-\gamma_{G3}\left[G3\right]$$ (3)

The rate of formation of Gal4p has basal expression and glucose repression term that modeled as Hill equation with Hill coefficient, *nG4* equal to 2.

$$\frac{d\left[G4\right]}{dt}=\alpha_{G4}\left(\frac{K_{R1}^{nG4}}{{\left[R\right]}^{nG4}+K_{R4}^{nG4}}\right)-k_{f84}\left[G4\right]\left[G80\right]+k_{r84}\left[C84\right]-\gamma_{G4}\left[G4\right]$$ (4)

The rate equation for GAL80 is given as,

$$\begin{array}{l} \frac{d\left[G80\right]}{dt}=\alpha_{0G80}+\alpha_{G80}\left(\frac{{\left[G4\right]}^{n80}}{{\left[G4\right]}^{n80}+K_{G80}^{n80}}\right)\left(\frac{K_{R80}^{nG80}}{{\left[R\right]}^{nG80}+K_{R80}^{nG80}}\right)-k_{f81}\left[G1*\right]\left[G80\right]+k_{r81}\left[C81\right]\\ -k_{f83}\left[G3*\right]\left[G80\right]+k_{r83}\left[C83\right]-k_{f84}\left[G4\right]\left[G80\right]+k_{r84}\left[C84\right]-\gamma_{G80}\left[G80\right] \end{array}$$ (5)

The above equation includes the production rate Gal80p with basal and induced expression, interaction with Gal1p, Gal4p, Gal3p, and the degradation of Gal80p. The active form of Gal1p (GAL1p*) has weak affinity towards Gal80p. The following equation gives the dynamics of Gal1p.

$$\frac{d\left[G1*\right]}{dt}=k_{f1}\left[G1\right]\left[B_{i}\right]-k_{r1}\left[G1*\right]-k_{f81}\left[G1*\right]\left[G80\right]+k_{r81}\left[C81\right]-\gamma_{G1p}\left[G1*\right]$$ (6)

The active form of Gal3p (Gal3p*) is formed as the result of galactose and Gal3p binding. Gal3p* interacts with Gal80p and form Gal80p-Gal3p* complex that prevents Gal80p Gal4p binding. The binding affinity between Gal3p* and Gal80p is stronger than Gal1p* and GAL80.

$$\frac{d\left[G3*\right]}{dt}=k_{f3}\left[G3\right]\left[B_{i}\right]-k_{r3}\left[G3*\right]-k_{f83}\left[G3*\right]\left[G80\right]+k_{r83}\left[C83\right]-\gamma_{G3p}\left[G3*\right]$$ (7)

The following equation describes the formation of intermediate complex molecules formed between Galp1*-Gal80p, Gal3p*-Gal80p, and Gal4p-Gal80p,

$$\begin{array}{l} \frac{d\left[C81\right]}{dt}=k_{f81}\left[G1*\right]\left[G80\right]-k_{r81}\left[C81\right]-\gamma_{C81}\left[C81\right]\\ \frac{d\left[C83\right]}{dt}=k_{f83}\left[G3*\right]\left[G80\right]-k_{r83}\left[C83\right]-\gamma_{C83}\left[C83\right]\\ \frac{d\left[C84\right]}{dt}=k_{f84}\left[G4\right]\left[G80\right]-k_{r84}\left[C84\right]-\gamma_{C84}\left[C84\right] \end{array}$$ (8)

### Transport Processes of Glucose and Galactose

The following equations describe the transport processes of glucose and galactose across the cell membrane. The rate change of the internal concentration of galactose molecules depends on the formation of biomass from galactose, the concentration of galactose transporter Gal2p and the formation of active protein Gal1p* and Gal3p*.

$$\frac{dB_{i}}{dt}=\frac{\lambda_{gal}}{G2_{max}}\left(\frac{\left[B_{e}\right]}{\left[B_{e}\right]+K_{\lambda B}}\right)\left[G2\right]-\left(\frac{\mu_{B,max}}{G1_{max}}\right)\left(\frac{\left[B_{i}\right]}{B_{i}+K_{B}}\right)\left[G1\right]\left[X\right]-k_{f3}\left[G3\right]\left[B_{i}\right]+k_{r3}\left[G3_{*}\right]-k_{f1}\left[G1\right]\left[B_{i}\right]+k_{r1}\left[G1_{*}\right]$$ (9)

The rate of intake of galactose molecules also depends on the concentration of Gal2 transporter protein.

$$\begin{array}{l} \frac{dB_{e}}{dt}=-\frac{\lambda_{gal}}{G2_{max}}\left(\frac{\left[B_{e}\right]}{\left[B_{e}\right]+K_{\lambda B}}\right)\left[G2\right]\left[X\right]\\ \frac{dA_{e}}{dt}=-\lambda_{glu}\left(\frac{\left[A_{e}\right]}{\left[A_{e}\right]+K_{\lambda A}}\right)\left[X\right] \end{array}$$ (10)

Here, A and B represent the glucose and galactose concentration respectively. The subscript *e* refers to extracellular concentration.

### Repression of Gal Network Through Mig1 Protein

The following equations are the rate equations for the Mig1p concentration (R). Mig1 protein becomes activated in presence of glucose.

$$\begin{array}{l} \frac{d\left[R\right]}{dt}=\alpha_{R}-k_{fR}\left[R\right]\left[A_{i}\right]+k_{rR}\left[Rp\right]-\gamma_{R}\left[R\right]\\ \frac{d\left[Rp\right]}{dt}=k_{fR}\left[R\right]\left[A_{i}\right]-k_{rR}\left[R_{p}\right]-\gamma_{Rp}\left[Rp\right] \end{array}$$ (11)

### Modelling of Mutant Strain

The rate equation of Gal3p for the *gal3*Δ mutant is modified as follow,

$$\begin{array}{l} \frac{d\left[G3\right]}{dt}=0\\ \frac{d\left[G3p\right]}{dt}=0 \end{array}$$ (12)

The epistatically altered strain was modelled by changing the appropriate parameter values in the model. The Gal3*-Gal80p interaction is weakened by a factor of 4, and the Gal80p-Gal4p interaction is weakened by a factor of 5, compared to the magnitude of these interactions in the ancestral strain.

The above equations are simulated simultaneously using Matlab ODE functions. Table 1 lists the values of the parameters used in the simulations.

**TABLE 1:** Parameters value used in both deterministic and stochastic model to simulate GAL network. Parameter values taken from (Venturelli et al., 2012), unless otherwise noted with a * in column 1.

Parameters	Value	Unit
$\alpha_{10}$ - GAL1 basal expression rate*	0.001	$nM.min^{-1}$
$\alpha_{80}$ - GAL80 basal expression rate	0.6	$nM.min^{-1}$
$\alpha_{30}$ - GAL3 basal expression rate*	0.001	$nM.min^{-1}$
$\alpha_{4}$ - GAL4 basal expression rate	0.5	$nM.min^{-1}$
$\alpha_{1}$ - GAL1 induced expression rate	15	$nM.min^{-1}$
$K_{G1}$ - GAL1 transcriptional feedback threshold	50	nM
$n_{1}$ - GAL1 Hill coefficient	2	no unit
$\alpha_{8}$ - GAL80 induced expression rate	0.9	$nM.min^{-1}$
$K_{G80}$ - GAL80 transcriptional feedback threshold*	20	nM
$n_{80}$ - GAL80 Hill coefficient	2	no unit
$\alpha_{3}$ - GAL3 induced expression rate	2	$nM.min^{-1}$
$K_{G3}$ - GAL3 transcriptional feedback threshold	50	nM
$n_{3}$ - GAL3 Hill coefficient	2	no unit
$k_{f1}$ - GAL1-galactose forward binding rate*	0.001	${\left(nM.\text{min}\right)}^{-1}$
$k_{f3}$ - GAL3-galactose forward binding rate*	1	${\left(nM.\text{min}\right)}^{-1}$
$k_{r1}$ - GAL1-galactose unbinding rate*	3	$min^{-1}$
$k_{r3}$ - GAL3-galactose unbinding rate*	10	$min^{-1}$
$k_{f81}$ - GAL1-GAL80 forward binding rate*	1	${\left(nM.\text{min}\right)}^{-1}$
$k_{f83}$ - GAL3-GAL80 forward binding rate*	1	${\left(nM.\text{min}\right)}^{-1}$
$k_{f84}$ - GAL4-GAL80 forward binding rate*	0.8	${\left(nM.\text{min}\right)}^{-1}$
$k_{r81}$ - GAL1-GAL80 unbinding rate*	0.1	$min^{-1}$
$k_{r83}$ - GAL3-GAL80 unbinding rate*	0.1	$min^{-1}$
$k_{r84}$ - GAL4-GAL80 unbinding rate*	1	$min^{-1}$
$\gamma_{G1}$ - GAL1 decay rate	0.004	$min^{-1}$
$\gamma_{G80}$ - GAL80 decay rate	0.004	$min^{-1}$
$\gamma_{G3}$ - GAL3 decay rate	0.004	$min^{-1}$
$\gamma_{G4}$ - GAL4 decay rate	0.004	$min^{-1}$
$\gamma_{G1p}$ - GAL1p decay rate	0.004	$min^{-1}$
$\gamma_{G3p}$ - GAL3p decay rate	0.004	$min^{-1}$
$\gamma_{C81}$ - Complex C81 decay rate	0.004	$min^{-1}$
$\gamma_{C83}$ - Complex C83 decay rate	0.004	$min^{-1}$
$\gamma_{C84}$ - Complex C84 decay rate	0.004	$min^{-1}$

### Stochastic Modelling of GAL Network

We use the Gillespie algorithm to implement the stochastic model for the GAL network (Gillespie, 1976). The dynamic reactions with their corresponding stochastic rates are listed in the Table 2. Galactose units are represented as A.U. in the model. These numbers closely match with the system’s response to galactose, when A.U. is replaced by nM. However, we note that different *S. cerevisiae* strains exhibit quantitatively different response to exposure to galactose (Lee et al., 2017). Hence, in this work, we focus on capturing the qualitative response of the system, to exposure to galactose.

**TABLE 2:** Reaction events and reaction rate used to simulate stochastic model.

Reaction ID	Reaction	Rate
R1: GAL1 basal expression	$\varphi \overset{\alpha_{10}}{\to }$ ***Gal1***	$\alpha_{10}$
R2: GAL80 basal expression	$\varphi \overset{\alpha_{80}}{\to }$ ***Gal80***	$\alpha_{80}$
R3: GAL3 basal expression	$\varphi \overset{\alpha_{30}}{\to }$ ***Gal3***	$\alpha_{30}$
R4: GAL4 basal expression	$\varphi \overset{\alpha_{4}}{\to }$ ***Gal4***	$\alpha_{4}$
R5: GAL1 expression by GAL4 induction	$\varphi \overset{\alpha_{1}}{\to }$ ***Gal1***	$\alpha_{1}\left(\frac{G4^{n1}}{G4^{n1}+K_{G1}^{n1}}\right)$
R6: GAL80 expression by GAL4 induction	$\varphi \overset{\alpha_{8}}{\to }$ ***Gal80***	$\alpha_{8}\left(\frac{G4^{n80}}{G4^{n80}+K_{G80}^{n80}}\right)$
R7: GAL3 expression by GAL4 induction	$\varphi \overset{\alpha_{3}}{\to }$ ***Gal3***	$\alpha_{3}\left(\frac{G4^{n3}}{G4^{n3}+K_{G3}^{n3}}\right)$
R8: GAl1 binding with galactose	$\mathrm{Gal}1+\mathrm{gal}\overset{k_{f1}}{\to }\mathrm{Gal}1_{p}$	$k_{f1}\left(G1\right)\left(gal\right)$
R9: GAL3 binding with galactose	$\mathrm{Gal}3+\mathrm{gal}\overset{k_{f3}}{\to }\mathrm{Gal}3_{p}$	$k_{f3}\left(G3\right)\left(gal\right)$
R10: Unbinding of GAL1-galactose	$\mathrm{Gal}1_{p}\overset{k_{r1}}{\to }\mathrm{Gal}1+\mathrm{gal}$	$k_{r1}\left(G1_{p}\right)$
R11: Unbinding of GAL3-galactose	$\mathrm{Gal}3_{p}\overset{k_{r3}}{\to }\mathrm{Gal}3+\mathrm{gal}$	$k_{r3}\left(G3_{p}\right)$
R12: Binding of GAL1p and GAL80	$\mathrm{Gal}1_{p}+\mathrm{Gal}80\overset{k_{f81}}{\to }\mathrm{Gal}81$	$k_{f81}\left(G1_{p}\right)\left(G80\right)$
R13: Binding of GAL3p and GAL80	$\mathrm{Gal}3_{p}+\mathrm{Gal}80\overset{k_{f83}}{\to }\mathrm{Gal}83$	$k_{f83}\left(G3_{p}\right)\left(G80\right)$
R14: Unbinding of GAL4-GAL80	$\mathrm{Gal}4+\mathrm{Gal}80\overset{k_{f84}}{\to }\mathrm{Gal}84$	$k_{f84}\left(G4\right)\left(G80\right)$
R15: Unbinding of GAL1p-GAL80	$\mathrm{Gal}81\overset{k_{r81}}{\to }\mathrm{Gal}1_{p}+\mathrm{Gal}80$	$k_{r81}\left(C81\right)$
R16: Unbinding of GAL3p-GAL80	$\mathrm{Gal}83\overset{k_{r83}}{\to }\mathrm{Gal}3_{p}+\mathrm{Gal}80$	$k_{r83}\left(C83\right)$
R17: Unbinding of GAL4-GAL80	$\mathrm{Gal}84\overset{k_{r84}}{\to }\mathrm{Gal}4+\mathrm{Gal}80$	$k_{r84}\left(C84\right)$
R18: decaying of GAL1	$\mathrm{Gal}1\overset{\gamma_{G1}}{\to }\theta$	$\gamma_{G1}\left(G1\right)$
R19: decaying of GAL80	$\mathrm{Gal}80\overset{\gamma_{G80}}{\to }\theta$	$\gamma_{G80}\left(G80\right)$
R20: decaying of GAL3	$\mathrm{Gal}3\overset{\gamma_{G3}}{\to }\theta$	$\gamma_{G3}\left(G3\right)$
R21: decaying of GAL4	$\mathrm{Gal}4\overset{\gamma_{G4}}{\to }\theta$	$\gamma_{G4}\left(G4\right)$
R22: decaying of GAL1p	$\mathrm{Gal}1_{p}\overset{\gamma_{G1_{p}}}{\to }\theta$	$\gamma_{G1_{p}}\left(G1_{p}\right)$
R23: decaying of GAL3p	$\mathrm{Gal}3_{p}\overset{\gamma_{G3_{p}}}{\to }\theta$	$\gamma_{G3_{p}}\left(G3_{p}\right)$
R24: decaying of Complex C81	$\mathrm{Gal}81\overset{\gamma_{C81}}{\to }\theta$	$\gamma_{C81}\left(C81\right)$
R25: decaying of Complex C83	$\mathrm{Gal}83\overset{\gamma_{C83}}{\to }\theta$	$\gamma_{C83}\left(C83\right)$
R26: decaying of Complex C84	$\mathrm{Gal}84\overset{\gamma_{C84}}{\to }\theta$	$\gamma_{C84}\left(C84\right)$

### Experimental Methods

The ancestor, epistatically-altered strain, and the *gal3*Δ strains used in this study are as described previously (Johnston and Hopper, 1982; Das Adhikari et al., 2014). To measure GAL1 promoter activity in these three strains GAL1-lacZ fusions were integrated at the *ura3* locus, as described previously (Das Adhikari et al., 2014).

### Growth Kinetics

Glycerol-lactate pre-grown strains were plated onto SCM agar plates (containing 2% glucose). The plates were thereafter incubated at 30°C for 2–3 days. Colonies were randomly selected from the agar plates and subjected to two rounds of serial passage in appropriate media [1% glucose, or 1% galactose, or glycerol/lactate (gly/lac)]. The resulting cultures were then washed with SCM and then growth curves were initiated with an initial optical density of 0.1 in SCM containing the appropriate carbon source (glucose, galactose, or gly/lac at the concentrations mentioned above). Three replicates of culture were transferred to a 96-well plate and OD was measured periodically until the cultures reach stationary phase. The plates were overlaid with a *Breathe Easy* membranes (Sigma) to prevent evaporation.

### GAL1 Expression Levels

GAL1-lacZ levels were determined in the three strains, ancestor, the epistatically altered strain, and the *gal3*Δ as described previously (Das Adhikari et al., 2014).

### Cell-Cell Heterogeneity (2-Deoxygalactose Experiments)

2DG-induced toxicity has been used previously to observe the metabolic state of a population (Platt, 1984; Das Adhikari et al., 2014). Cells were inoculated into gly/lac medium and incubated at 30 deg C for 48–72 h with shaking. These gly/lac pregrown cultures were inoculated into 5 ml of CSM containing 1% galactose, such that the starting OD of the culture was equal to 0.01. Cells were harvested from this growing culture, serially diluted with PBS and thereafter, plated onto gly/lac solid media containing 2-Dexoygalactose (2DG) (0.3 μM). As a control, cells were also plated on media with gly/lac and no 2DG. The two sets of plates were incubated at 30 deg C for 3–4 days. The number of colonies that grew on gly/lac plates and those containing 2DG were counted and the percentage of Gal-positive cells calculated. All experiments were repeated three times. A minimum of 500 colonies on gly/lac plates were counted in each experiment.

## Results

### Experimental Characterization of the GAL Regulon/Network in Wild-Type and Mutant *S. cerevisiae*

We first start by experimentally characterizing the growth kinetics of cells in a galactose medium. In this work, we focus on three different strains. First, the ancestral wild type; second, an epistatically-altered strain in which the Gal3*-Gal80p interaction is weakened by a factor of four, and the Gal80p-Gal4p interaction is weakened by a factor of five (Das Adhikari et al., 2014). These changes in the interaction strengths are due to the strain having mutant alleles of Gal80p and Gal4p.

**FIGURE 2:**
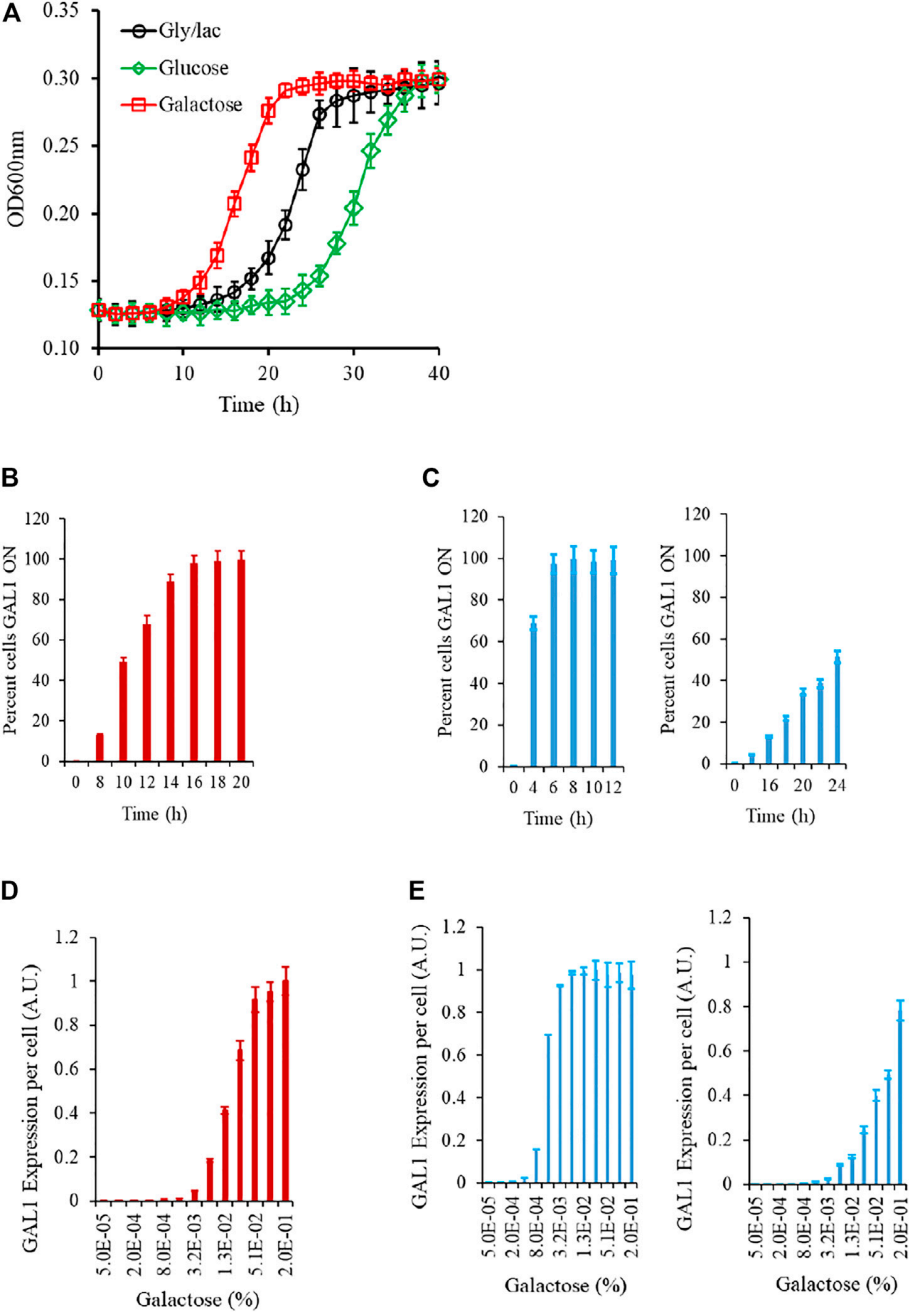
Experimental characteristics of the GAL network in *S. cerevisiae.* **(A)** Growth kinetics in galactose is contingent on the conditions in which the cells are introduced from. The lag is the longest in the cells introduced from glucose, and shortest in those introduced from galactose. **(B)** During transition from gly/lac to galactose, the percent cells which are expressing GAL1 is heterogeneous in the initial phase of the growth. **(C)** Same as **(B)**. The transition from lag to log phase in the epistatically-altered strain (left) and the *gal3*Δ strain (right) is qualitatively different. **(D)** Steady state GAL1 expression in ancestral strain, when cells are grown in different galactose concentrations. **(E)** Same as **(C)** but for the epistatically-altered strain (left) and the *gal3*Δ strain (right). The nature of galactose gene expression in the three strains is qualitatively different from each other. All experiments were performed in triplicate. The average and standard deviations are reported.

We first study the growth kinetics of the wild type strain, as the cells are brought to a galactose environment from the same/different carbon source environment. As shown in the Figure, the populations exhibits a lag phase duration which is a function of the environment from which the cells are brought into the galactose media. At a single-cell resolution, the transition from a GAL OFF to a GAL ON state is heterogeneous; and the wild type strain exhibits a characteristic gene expression levels, when exposed to a certain galactose concentration. (Figure 2; Supplementary Figure S1). As shown in the Figure, as cells are brought to a media containing galactose, the initial lag phase duration is a function of the environment the cells are brought from. The lag phase is the longest when cells are brought from repressing conditions (glucose), medium when brought from non-inducing non-repressing (NINR) conditions (glycerol/lactate), and shortest when brought from inducing conditions (galactose). In the time region when the cells transition from lag to log phase, the single-cell behavior of the gene expression levels was characterized. For this purpose, we withdrew cells at regular intervals, and plated an equal volume on gly/lac plates, and on gly/lac plates containing 2DG. All cells expressing Gal1p only grow on the gly/lac plates. This assay thus allows us to quantify, at a single-cell resolution, how cells transition from a GAL OFF state to a GAL ON state. As shown in the Figure, the wild-type strain exhibits a heterogeneous induction kinetics in the window of transition from lag to log phase. On the other hand, the epistatically-altered mutant exhibits a kinetics where the induction is homogeneous and faster, as compared to the wild type. On the other hand, in the *gal3*Δ, the induction kinetics are considerably slower, and heterogeneous, even at long durations of time. Note that in the *gal3*Δ, the signal transduction activity is carried out by the kinase, Gal1p, which is a bifunctional protein is capable of weak signal transduction activity also (Meyer et al., 1991).

The steady state Gal1p levels, when studied via a proxy of a promoter fusion reporter indicate that GAL genes are turned ON, when the concentration of galactose in the media exceeds a threshold. These thresholds and the nature of induction are qualitatively different in the three strains being analyzed in this study. Thus, we establish that the nature of response to a galactose environment in *S. cerevisiae* is strongly influenced by the protein-protein interactions in the regulatory network.

### GAL Regulon Model Captures the Kinetics of Gene Expression

#### Kinetics of GAL System Induction are Strongly Dependent on the Initial Conditions

**FIGURE 3:**
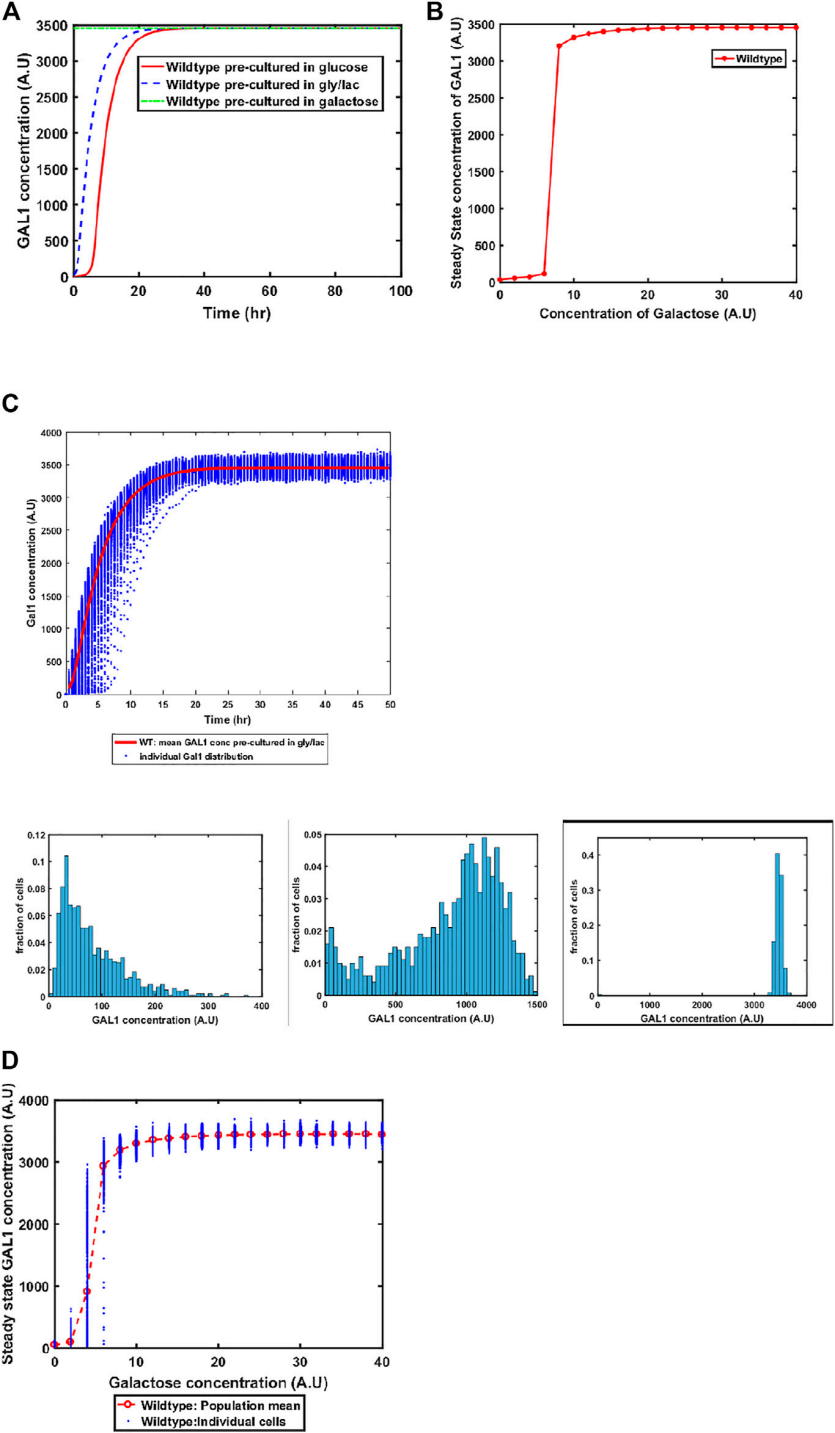
**(A)** Activation of GAL network in galactose [concentration, 30 (A.U)]. GAL1 profile of wildtype pre-cultured in gly/lac (dashed blue line), glucose (solid red line), and galactose (dashed green line). **(B)** Steady state concentration of GAL1 protein at different concentrations of galactose in wild type *S. cerevisiae*. Note that the system exhibits a threshold-like activation. **(C)** Dynamic GAL1 response at galactose concentration of 30 (A.U) for 1000 wild-type cells pre-grown in gly/lac. Histograms of GAL1 expression at 30 min, 5 h, and 50 h. **(D)** Steady state concentration of GAL1 under different concentration of galactose for wild type.

The GAL system in *S. cerevisiae* is induced in the presence of galactose, and is actively repressed in the presence of glucose. On the other hand, the system is in a Non-Induced Non-Repressed conditions (NINR), when cells are grown in gly/lac media. This aspect of the system is captured in the model (Figure 3A). When cells are transferred to a media containing galactose, the duration of the lag phase is contingent on the pre-existing state of the system. The lag phase is the longest in the cells which are introduced into galactose-containing media from glucose, and is shortest in the cells which come from galactose.

#### Switch-Like Induction of GAL Genes

Next, we simulate the steady state concentration of GAL1 levels when cells are grown in different galactose concentrations. As shown in the Figure 3B, GAL gene induction follows a step-like behavior. For small concentrations of galactose in the media, the increase in GAL gene induction is small and linear in nature. Beyond a threshold, however, the GAL1 gene expression levels increases in a step function manner to their maximal levels. Thereafter, the steady state concentrations of GAL1 do not change with further increase in galactose.

#### Positive Feedback, and Cell-Cell Heterogeneity in the GAL System

Sugar utilization systems are a combination of positive and negative feedback loops. The GAL4p-encoded positive feedback leads to rapid induction of the system. As cells transition from the GAL OFF to a GAL ON state, there is cell-cell heterogeneity in the system (Figure 3C). At intermediate times, a fraction of the cells are in the ON state, and the rest of the population is in the OFF state. This heterogeneous induction kinetics are characteristic of sugar utilization systems in bacteria. At a steady state level, the GAL network exhibits cell-cell heterogeneity at steady state in low concentrations of galactose (Figure 3D).

### Altering the GAL Network Architecture

Two aspects of the GAL network stand out. The first, GAL system in *S. cerevisiae* comprises of GAL1 and GAL3, two genes which resulted from a whole genome duplication event (Marcet-Houben and Gabaldon, 2015; Wolfe, 2015). Gal3p is a signal transducer, which dictates the kinetics of the system; and Gal1p is a galactokinase, which also has limited signal transduction activity. Therefore, a *gal1*Δ strain cannot grow on galactose, whereas a *gal3*Δ strain can exhibit growth on galactose. Second, ecological isolates of *S. cerevisiae* exhibit a wide range of growth kinetics, when grown on a mixture of glucose and galactose. The glucose-dependent repression of the GAL system is strain specific, and as a result, the transition from glucose to galactose varies from strain to strain. Interestingly, this behavior was found to be largely dictated by the allelic variation at the GAL3 locus (Lee et al., 2017). In a recent study, evolution on melibiose was studied, and interestingly, alternate GAL3 alleles were reported discovered (Anjali et al., 2021). Additionally, alternate GAL80 alleles were isolated which exhibit different kinetics of induction, when introduced to a galactose environment (Nogi and Fukasawa, 1984; Salmeron et al., 1990; Douglas and Hawthorne, 1966). This change in kinetics is due to the altered GAL80 allele exhibiting altered binding behavior with GAL4 and GAL3* (GAL3* is GAL3 bound to galactose). Alternate alleles have been isolated in short term evolution experiments. We next use the model to study these two aspects of the network. We mimic the *gal3*Δ mutant by putting GAL3 amounts in the cell equal to zero at all times. We mimic alternate GAL3 alleles by changing the strength of GAL3 interaction with GAL80.

#### Epistatically-Altered Strain of the GAL Network

**FIGURE 4:**
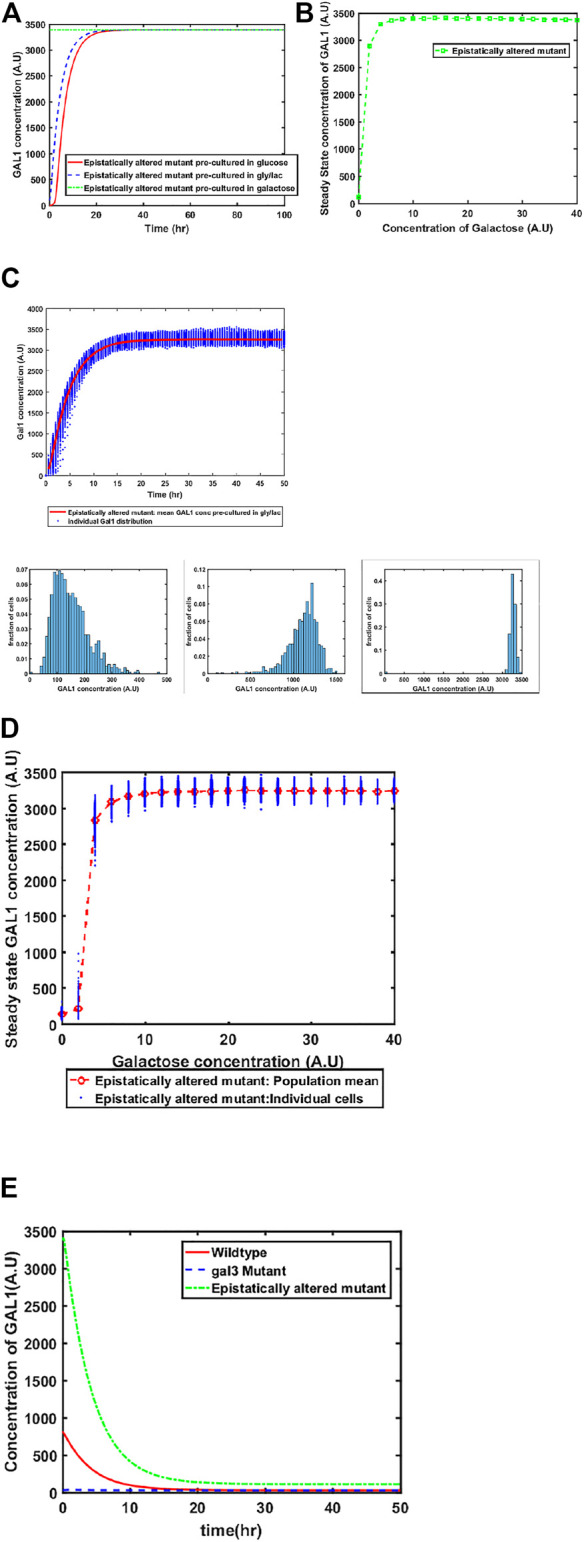
Dynamics of gene expression for the epistatically-altered strain. **(A)** Activation of GAL network in galactose [concentration, 30 (A.U)]. GAL1 profile when cells are pre-cultured in gly/lac (dashed blue line), glucose (solid red line), and galactose (dashed green line). **(B)** Steady state concentration of GAL1 protein at different concentrations of galactose. Note that the system exhibits a behaviour which is qualitatively different than the wild type behavior. The threshold of galactose concentration is no longer present. **(C)** Dynamic GAL1 response at galactose concentration of 30 (A.U) for 1000 cells pre-grown in gly/lac. Histograms of GAL1 expression at 30 min, 5 h, and 50 h. **(D)** Steady state concentration of GAL1 under different concentration of galactose. **(E)** The dynamics of transition from ON to OFF state in the ancestor and the epistatically altered strain. Note that even after long times, the epistatically altered strain has a non-zero residual GAL1 expression. The GAL1 profile for the *gal3*Δ is shown as control.

Mutant of GAL4 which exhibited constitutive GAL network induction was isolated a long time back. This mutant allele (GAL4^c^) does not interact with GAL80, and thus, cells containing this altered allele have the GAL network in the ON state, independent of the environmental conditions. To restore galactose-dependent induction of the GAL network, Mutants of GAL80 were isolated (e.g., GAL80^s−1^). The GAL80^s−1^ allele interacts with GAL4^c^, and thus restores galactose-dependent induction. However, the mutant alleles (GAL4^c^ and GAL80^s−1^) exhibit altered binding behavior between Gal3*-Gal80p, Gal1*-Gal80p, and Gal80p-Gal4p. The biochemical interactions between Gal4^c^-Gal80^s−1^ and that between Gal80^s−1^-Gal3* have been previously characterized (Das Adhikari et al., 2014). When we make these changes in the model to reflect altered binding kinetics, we predict that the behavior of the system at a 1) population level, and 2) single-cell resolution changes from that of the wild type. As shown in the Figure, our model predicts that the epistatically-altered strain exhibits 1) faster induction of the GAL system, 2) higher steady state levels of the GAL proteins, 3) a qualitatively different induction kinetics at a single-cell resolution, and 4) a slower transition from the ON to the OFF state, when cells are moved from a galactose- to a glucose-containing media (Figure 4).

#### GAL3 Mutant Exhibits Altered Gene Expression Kinetics, Including Bistability at Intermediate Galactose Concentrations

**FIGURE 5:**
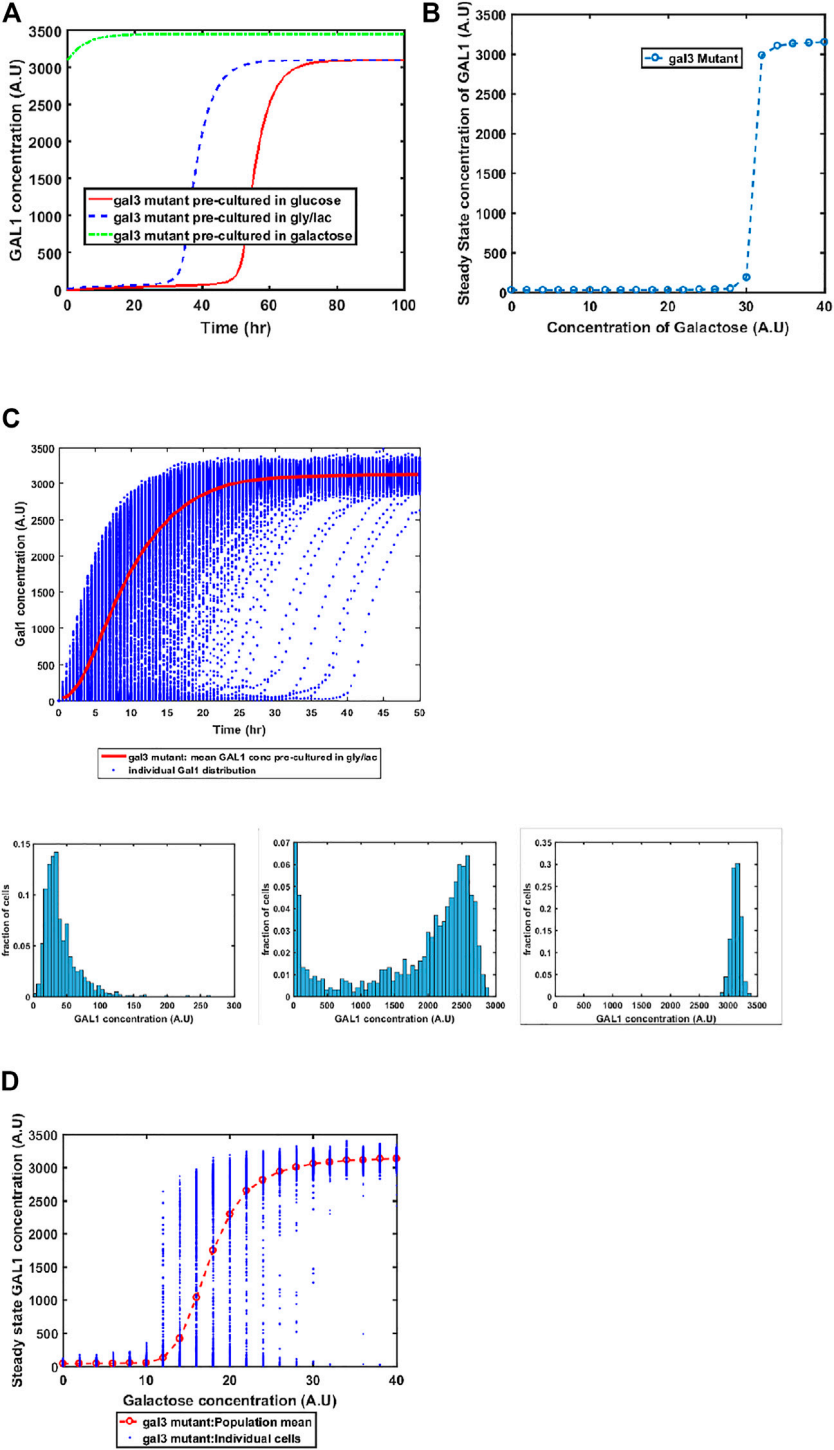
Dynamics of gene expression for *gal3*Δ strain. **(A)** Activation of GAL network in galactose [concentration, 30 (A.U)]. GAL1 profile when cells are pre-cultured in gly/lac (dashed blue line), glucose (solid red line), and galactose (dashed green line). **(B)** Steady state concentration of GAL1 protein at different concentrations of galactose. Note that the system exhibits a behaviour which is qualitatively different than the wild type behavior. The threshold of galactose concentration is no longer present. **(C)** Dynamic GAL1 response at galactose concentration of 30 (A.U) for 1000 cells pre-grown in gly/lac. Histograms of GAL1 expression at 30 min, 5 h, and 50 h. **(D)** Steady state concentration of GAL1 under different concentration of galactose.

GAL1 and GAL3 are homologous genes, resulting from a whole genome duplication event about a 100 million years ago (Marcet-Houben and Gabaldon, 2015; Wolfe, 2015). The ancestral gene sequence presumably had both kinase and the signal transduction activity. Since duplication and divergence, Gal3p has lost the kinase activity in *S. cerevisiae* and has evolved to become a better signal transducer than the ancestor. On the other hand, Gal1p has evolved to become a specialist galactokinase, while still retaining small signal transduction activity. This adaptive divergence is thought to have resolved an adaptive conflict where one gene sequence was coding for two activities (Hittinger and Carroll, 2007). Since Gal3p does not have any kinase activity, a *gal1*Δ does not exhibit any growth in galactose. On the other hand, a GAL3Δ exhibits growth, albeit with delayed kinetics, when grown in the presence of galactose. Our model successfully captures this facet of growth on galactose (Figure 5). Loss of GAL3 manifests in not only lower Gal1p activity, but also the switch-like induction of the GAL regulon takes place at a significantly higher concentration. At a single-cell resolution, a *gal3*Δ strain exhibits exaggerated heterogeneity as the population transitions from a GAL OFF to a GAL ON state. In addition, steady state levels of Gal1p also exhibit bistability at intermediate concentrations of galactose. Note that this bistability was not observed in the ancestral strain (Figure 2), or in the epistatically-altered strain (Figure 3). Thus, a *gal3*Δ strain exhibits significantly altered growth kinetics, both at a population level, and at a single-cell resolution.

**FIGURE 6:**
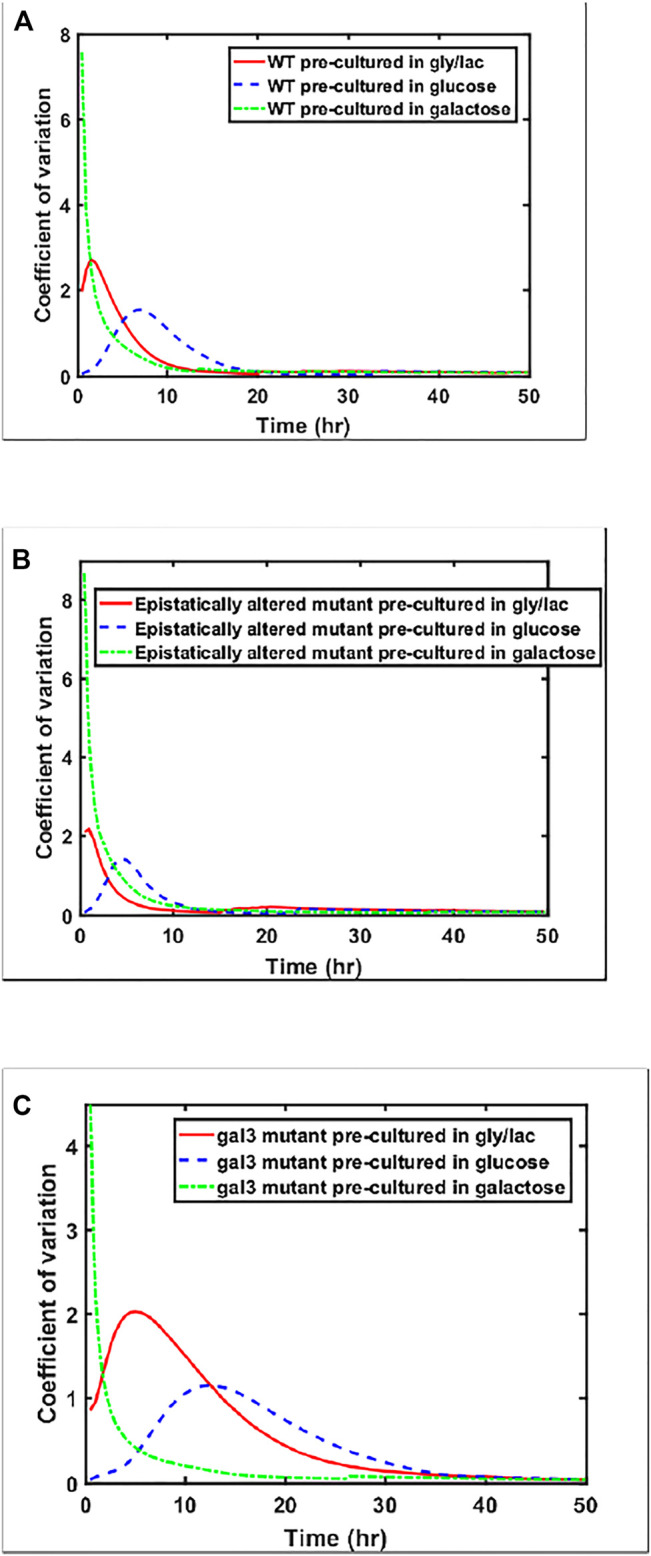
Coefficient of variation in GAL1 expression levels, shown for **(A)** wild type, **(B)** Epistatically-altered strain, and **(C)** *gal3*Δ strain when grown in different environmental conditions.

While the steady state response of the three strains is within a factor of 1.2 for the three strains examined above (ancestor, epistatically-altered strain, and *gal3*Δ), the kinetics of gene expression vary widely. This difference can also be seen from analyzing the coefficient of variation between the expression levels of the members of the populations as cells are transitioned from a given culture conditions to galactose (Figure 6). Cell-cell heterogeneity among isogenic populations is now known to manifest itself in a number of ways—often providing adaptive value to the population. Hence, from the prospect of a population to move towards higher fitness levels, the cell-cell variability in a population is an evolutionarily important marker.

### Evolution *via* Changes in the Coding Regions of the Regulatory Genes of the GAL Network

**FIGURE 7:**
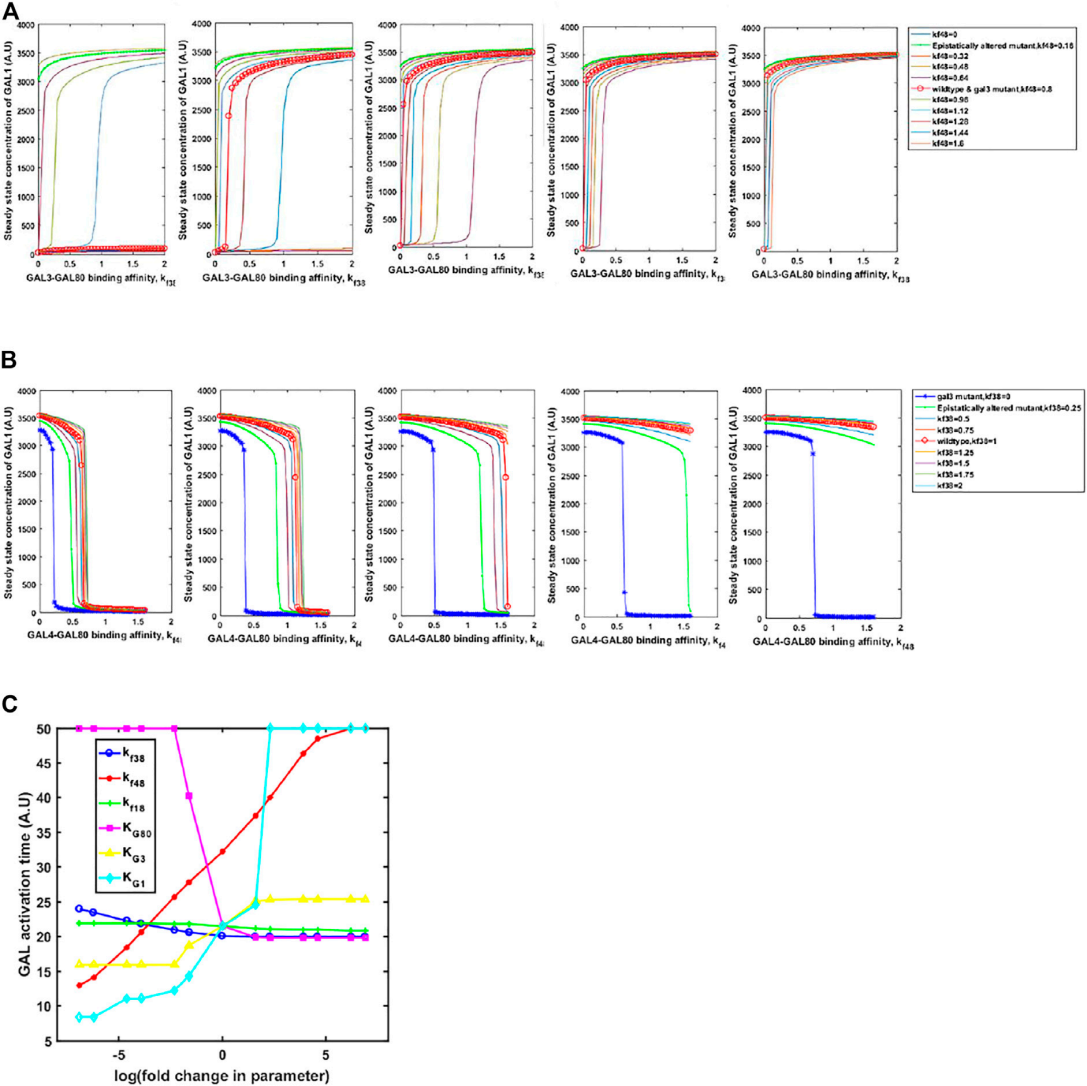
Steady state GAL1 concentration plotted against the binding affinity between GAL3-GAL80 for different GAL4-GAL80. In **(A)** the change in GAL3-GAL80 interaction is represented on the *x*-axis. Different curves represent different strengths of GAL4-GAL80 interaction, as indicated in the legend. In **(B)**, the change in GAL4-GAL80 interaction is represented on the *x*-axis. Different curves represent different strengths of the GAL3-GAL80 interaction, as indicated in the legend. In both **(A)** and **(B)**, the five panels represent a galactose concentration of 5, 10, 15, 20, and 25 A.U. (from left to right). **(C)** Time taken to reach maximal GAL1p production rate, as cells transition from glucose to galactose.

Adaptive changes are largely studied as a result of changes in gene expression, which are obtained via changes in one or more of the following: promoter activity, mRNA stability, translation rate. In all these mutations, adaptive benefit takes place as a result of changes in expression levels of the protein. However, as mentioned above, a large number of alleles of GAL regulatory genes, with altered biochemical interactions, have been identified in both, laboratory studies as well as analysis of ecological isolates (Peng et al., 2015; Roop et al., 2016; Lee et al., 2017). These alleles confer qualitatively different growth dynamics, depending on the environmental context. Thus, we hypothesize that the population level and the single-cell behavior of the GAL network can be tuned to a large extent *via* changes in the protein coding sequence which change the protein function rather than levels. This is indicated in Figures 7A, B. On changing the interaction strengths between the Gal3*-Gal80p and Gal4p-Gal80p binding propensities, the steady-state induction levels of Gal1p levels are highly tunable. This tuning of gene expression is achieved by simply changing one/two binding coefficients between the regulatory proteins. On the other hand, if the same change made in the *gal3*Δ, the corresponding change in Gal1p protein levels are not observed. Note that a *gal3*Δ strain is able to exhibit growth in galactose, because its regulatory function is compensated by the signal transduction activity of Gal1p.

On the other hand, kinetics of growth on glucose and galactose are also strongly influenced by the parameters. We examine the change in the diauxy lag when cells growing on glucose are introduced to a media containing galactose. As shown in Figure 7C, the duration of lag is highly tuned by changing the affinity of protein-protein interactions in the regulatory network, or by changing the half-maximum concentration of the proteins. Interestingly, our simulations show that changing the GAL3p-GAL80p (GAL1p-GAL80p) interaction strength has the least effect on the duration of the lag phase. This is in contrast to changing the interaction strength of GAL4p-GAL80p, which controls the lag phase duration much more strongly. The duration of the lag phase as yeast transition from glucose to galactose has been attributed to several genetic loci (Peng et al., 2015; Roop et al., 2016; Lee et al., 2017), thus indicating a distributed control strategy. Among these interactions, our results demonstrate that GAL4p-GAL80p interaction is the key major determinant of the lag-phase duration, as cells transition from glucose to galactose; and the GAL3p-GAL80p is a fine-tuning interaction of the lag phase duration during this transition.

## Discussion and Conclusion

The GAL system in the yeast *S. cerevisiae* is, along with the lac system in *E. coli*, perhaps the most well studied and well characterized gene regulation and sugar utilization systems. Interestingly, while the lac system has been studied in thousands of reports, our understanding of allelic variation resulting in changes in kinetics or levels of gene expression are limited. On the other hand, several alleles of regulators are known. The reasons for this difference are not clear.

*S. cerevisiae* exists primarily as a diploid in its ecological niches. In fact, several laboratory experiments have conclusively demonstrated that propagation of a haploid for a few hundred generations leads to self-diploidization (Gerstein et al., 2006; Venkataram et al., 2016; Harari et al., 2018). This process is accelerated in conditions of stress (Gerstein et al., 2006). Presumably, doubling the genome size allows for a faster adaptation to the prevailing stressful conditions. In the context of the GAL network, *S. cerevisiae* has several mutational targets to change the kinetics of gene expression. These include the signal transducer GAL3, the repressor GAL80, the transcriptional activator GAL4, and the galactokinase (which has small regulatory activity) GAL1. This diversity in the mutational targets, presumably allows the organism to tune gene expression and physiology which is most appropriate for the surrounding environmental conditions. This is also suggested by a recent analysis of the GAL network, where specific combinations of regulatory alleles were found to confer high fitness; and others found to lead to unfit individuals (Boocock et al., 2021). In fact, analysis of the GAL network from several experimental studies has indicated that the tuning of the GAL gene expression is primarily controlled by modifications in the regulatory proteins, and not by changes in the promoter regions driving expression of the GAL enzymes or regulatory proteins (Wang et al., 2015; Lee et al., 2017).

Towards this end, recent evidence has indicated that mutations in regulatory proteins are likely significant contributors of gene expression evolution (Brem et al., 2002; Yvert et al., 2003; Bustamante et al., 2005). Interestingly, among these mutations, gene duplication is an important driver of changing regulatory behavior in a gene network (Voordeckers et al., 2012; Baker et al., 2013; Perez et al., 2014; Pougach et al., 2014), resulting in GAL1 and GAL3. Gene duplication and the consequent divergence has been suggested to be involved in ecological adaptation in yeast, in another context (Thomson et al., 2005).

Comparing the GAL network with well-characterized sugar utilization systems in bacteria, we note that the GAL network in yeast is distinct in its structure. While most sugar utilization systems in *E. coli* employ only one regulaor (AraC for arabinose; XylR for xylose; LacI for lactose) (Aidelberg et al., 2014) [however, one exception, in the form of rhamnose utilization system does exist (Kolin et al., 2008)], the GAL system employs a cascade of three. We speculate that the additional number of regulators in the GAL network help individual strains to adapt to their precise ecological niche. Evolution experiments to test the relationship between complexity of regulatory structures and adaptive response of populations can shed more light on this aspect of cellular physiology and adaptation.

## Data Availability

The datasets presented in this study can be found in online repositories. The names of the repository/repositories and accession number(s) can be found in the article/Supplementary Material.
