# Supplementary material for: GAL Regulon in the Yeast S. cerevisiae is Highly Evolvable via Acquisition in the Coding Regions of the Regulatory Elements of the Network
Source: Front Mol Biosci. 2022 Mar 14;9:801011. doi: 10.3389/fmolb.2022.801011 (PMC8964464; doi:10.3389/fmolb.2022.801011)
Supplement: Supplementary file 2 [file DataSheet1.PDF]

Code.

MATLAB code for deterministic model

```
clc;
clear all;
close all;

global  alphaG10 alphaG30 alphaG20 alphaGM0 alphaG1 n1 KG1 kf81 kr81 gammaG1
alphaG3 n3 KG3...
    kf3 kr3 kf83 kr83 kf1 kr1 gammaG1p gammaG3 gammaG3p alphaG4 kf84 kr84
gammaG4 alphagal0G80 alphagalG80 n80 KG80 ...
    gamaG80 gammaC81 gammaC83 gammaC84 KA KB alphaGM alphaG2 lam_M gammaGM
gammaG2...
    Vmax KM KG2 KGM mu_Amax mu_Bmax lam_glu lam_gal nM n2 KAB KBB KAR1 KAR4
KAR3...
    n1G  n3G  nA nB nMel kd n4G nAt nBt  gal1max gal2max alphaR kfR krR
gammaR gammaRp mel1max;

%Parameter set I in secs
%Basal Expressions
alphaG10=0.001;
alphaG30=0.001;
alphaG20=0.001;
alphaGM0= 0.001;
alphagal0G80=0.7;
%Induced Expression
alphaG1=15;
alphaG2=2;
alphaG3=2;
alphaGM=0.5;
alphagalG80=1;
alphaG4=0.84 ;
alphaR=1;

%Hill coefficients
n1=3;
n3=2;
n80=2;
nM=1;
n2=2;
n1G=2;
n3G=2;
nA=1;
nB=1;
n4G=1;
nAt=1;
nBt=1;
nMel=1;
%Binding affinity
KG1=50;
KG3=50;
KG80=20;
KA=1;
KB=1;
KM=1;
KG2=20;
KGM=10;
KAB=1;
```

```
KBB=1;
KAR1=10;
KAR4=10;
KAR3=10;
kd=1;
%Interaction coefficient
kf1=0.001;
kr1=3;
kf3=0.1;
kr3=10;

kf81=1;
kr81=0.1;
kf83=1;
kr83=0.1;

kf84=0.8;
kr84=1;

kfR=10;
krR=200;



%Decay rate
gamma=0.004;
gammaG1=gamma;
gammaG1p=gamma;
gammaG3=gamma;
gammaG3p=gamma;
gammaG4=gamma;
gamaG80=gamma;
gammaC81=gamma;
gammaC83=gamma;
gammaC84=gamma;
gammaGM=gamma;
gammaG2=gamma;
gammaR=gamma;
gammaRp=gamma;
%Transport Property
lam_M=0.5;
lam_glu=0.2;
lam_gal=0.2;
%Specific Growth rates
Vmax=0.1;
mu_Amax=0.5/60;
mu_Bmax=0.2/60;
%Normalizing values
gal1max=110;
gal2max=10;
mel1max=20;
%%
tspan = 0:0.05:3000;
opts = odeset('NonNegative',1:10);
X1=[0 0 0 0 0 0 0 0 0 0]'; % initial condition

gal=0:2:40;
for i=1:1:length(gal)
[t1m,Y1] = ode15s(@(t,X) onlyGAL(t,X,(gal(i))),tspan,X1,opts);
```

```
t1=t1m;
ssgal1w(i)=Y1(end,1);%+Y1(end,7)+Y1(end,10);
ssgal3w(i)=Y1(end,3)+Y1(end,6)+Y1(end,8);
ssgal4w(i)=Y1(end,4);
% figure(1)
% plot(t1,Y1(:,1))
end

%%
for i=1:1:length(gal)
[t2m,Y2] = ode15s(@(t,X) noGAL3(t,X,(gal(i))),tspan,X1,opts);
t2=t2m;

ssgal1m(i)=Y2(end,1);%+Y2(end,7)+Y2(end,10);
ssgal3m(i)=Y2(end,3)+Y2(end,6)+Y2(end,8);
ssgal4m(i)=Y2(end,4);
% figure(2)
% plot(t2,Y2(:,1))
end
%%
kf83=1/4;

kf84=0.8/5;

for i=1:1:length(gal)
[t3m,Y3] = ode15s(@(t,X) onlyGAL(t,X,(gal(i))),tspan,X1,opts);
t3=t3m;

ssgal1m2(i)=Y3(end,1);
ssgal3m2(i)=Y3(end,3)+Y3(end,6)+Y3(end,8);
ssgal4m2(i)=Y3(end,4);
% figure(2)
% plot(t2,Y2(:,1))
end

%%

% figure
% subplot(1,3,1)
% plot(gal,ssgal1w,'*-',gal,ssgal1m,'o--',gal,ssgal1m2,'g--');
% xlabel('Concentration of Galactose (A.U)');
% ylabel('Steady State concentration of GAL1total (A.U)')
% subplot(1,3,2)
% plot(gal,ssgal3w,'*-',gal,ssgal3m,'o--',gal,ssgal3m2,'g--');
% xlabel('Concentration of Galactose (A.U)');
% ylabel('Steady State concentration of GAL3total (A.U)')
% subplot(1,3,3)
% plot(gal,ssgal4w,'*-',gal,ssgal4m,'o--',gal,ssgal4m2,'g--');
% xlabel('Concentration of Galactose (A.U)');
% ylabel('Steady State concentration of GAL4 (A.U)')
plot(gal,ssgal1w,'*r-',gal,ssgal1m,'o--',gal,ssgal1m2,'square--g');
xlabel('Concentration of Galactose (A.U)');
ylabel('Steady State concentration of GAL1total (A.U)')
legend('Wildtype','gal3 Mutant','Epistatically altered mutant')
```

Function file

```
function Y=onlyGAL(t,X,gal)
Y=zeros(10,1);
```

```
global  alphaG10 alphaG30 alphaG20 alphaGM0 alphaG1 n1 KG1 kf81 kr81 gammaG1
alphaG3 n3 KG3...
    kf3 kr3 kf83 kr83 kf1 kr1 gammaG3 gammaG1p gammaG3p alphaG4 kf84 kr84
gammaG4 alphagal0G80 alphagalG80 n80 KG80 ...
    gamaG80 gammaC81 gammaC83 gammaC84 KA KB alphaGM alphaG2 lam_M gammaGM
gammaG2...
    Vmax KM KG2 KGM mu_Amax mu_Bmax lam_glu lam_gal nM n2 KAB KBB KAR1 KAR4
KAR3...
    n1G  n3G  nA nB nMel kd n4G nAt nBt  gal1max gal2max alphaR kfR krR
gammaR gammaRp mel1max;

%Gal Network inside each Cell
%Gal1
Y(1)= alphaG10 + alphaG1*((X(4)^n1)/((X(4)^n1)+((KG1)^n1))) + kr1*X(10)-
kf1*X(1)*gal-(gammaG1)*X(1);
%Gal2
Y(2)= alphaG20+ alphaG2*((X(4)^n2)/((X(4)^n2)+((KG2)^n2)))- (gammaG2)*X(2);
%Gal3 inactive
Y(3)= alphaG30 + alphaG3*((X(4)^n3)/((X(4)^n3)+((KG3)^n3))) - kf3*X(3)*gal +
kr3*X(6) - (gammaG3)*X(3);
%Gal4
Y(4)= alphaG4 - kf84*X(4)*X(5) + kr84*X(9) - (gammaG4)*X(4);
%Gal80
Y(5)= alphagal0G80 + alphagalG80*((X(4)^n80)/((X(4)^n80)+((KG80)^n80)))...
    - kf81*X(10)*X(5)+ kr81*X(7)- kf83*X(6)*X(5)+ kr83*X(8) - kf84*X(4)*X(5)
+ kr84*X(9) - (gamaG80)*X(5);
%Gal3p active
Y(6)= kf3*X(3)*gal - kr3*X(6) - kf83*X(6)*X(5)+ kr83*X(8)-(gammaG3p)*X(6);
%activated Gal1p
Y(10)=kf1*X(1)*gal- kr1*X(10)- kf81*X(10)*X(5)+ kr81*X(7)-(gammaG1p)*X(10);
%Complex81
Y(7)= kf81*X(10)*X(5)- kr81*X(7)- (gammaC81)*X(7);
%Complex83
Y(8)= kf83*X(6)*X(5)- kr83*X(8)- (gammaC83)*X(8);
%Complex84
Y(9)= kf84*X(4)*X(5) - kr84*X(9) - (gammaC84)*X(9);
end
```

C - code for stochastic simulation

```
#include<stdio.h>
#include<stdlib.h>
#include<math.h>
#include<memory.h>
#include<time.h>
#include<conio.h>
#include<process.h>
#include<dir.h>
#include <unistd.h>

// Mean value of a vector
float mean(float var[],int Size)
{
        float sum=0;

        for(int i=0;i<Size;i++)
        {
                sum=sum+var[i];
        }
        return (float)sum/Size;
}
float sumn(float var[],int Size)
{
        float sum=0;

        for(int i=0;i<Size;i++)
        {
                sum=sum+var[i];
        }
        return (float)sum;
}

int main()
{
        srand(time(0));
        //get current time
        // to store execution time of code
        double time_spent = 0.0;
        clock_t begin = clock();
        int galc[21]={0,2,4,6,8,10,12,14,16,18,20,22,24,26,28,30,32,34,36,38,40};
        char dirname1[500];
        sprintf(dirname1, "gal1000para%d",(rand() % ((1000) - 0 + 0)) + 0);
        mkdir(dirname1);
for (int  exp = 0; exp < 21; exp++)
{

        FILE *fp=NULL;
        //Create file for steady state
        FILE *SS=NULL;
        char filenameSS[500];

                        sprintf(filenameSS, "SSgal%d.dat",galc[exp]);

                        char filePathSS[500];

                        sprintf(filePathSS, "%s/%s",dirname1,filenameSS);


                        SS=fopen(filePathSS,"a");

for (int  run = 0; run < 1000; run++)
{

    int gal=galc[exp];
    printf("Expriment run: %d with Galactose = %d numbers....\n",run,gal);
    float maxt=3000;
```

```
// Parameter set I
// Basal Expressions
float alphaG10=0.001;
float alphaG30=0.001;
//GAL3 Knockout
//float alphaG30=0.00;
float alphaGM0= 0.001;
float alphagal0G80=0.6;
// Induced Expression
float alphaG1=15;
float alphaG3=2;
//GAL3 Knockout
//float alphaG3=0;
float alphagalG80=0.9;
float alphaG4=0.5;

// Hill coefficients
int n1=2;
int n3=2;
int n80=2;

// Binding affinity
int KG1=50;
int KG3=50;
int KG80=20;

// Interaction coefficient
float kf1=0.001;
float kr1=3;
float kf3=1;
float kr3=10;

float kf81=1;
float kr81=0.1;
float kf83=1;
float kr83=0.1;

float kf84=.8;
float kr84=1;

// Decay rate
float gamma=0.004;

 float w[23]={0,0,0,0,0,0,0,0,0,0,0,0,0,0,0,0,0,0,0,0,0,0,0};


    int X[9]={0,0,0,0,0,0,0,0,0};

    // %X(0) = Gal1
    // %X(1) = Gal80
    // %X(2) = Gal3
    // %X(3) = Gal4
    // %X(4) = Gal1p
    // %X(5) = Gal3p
    // %X(6) = C81
    // %X(7) = C83
    // %X(8) = C84

// Calculating Propensity function
    // Gal protein production (basal + induced)
    w[0]=alphaG10 + alphaG1*(pow(X[3],n1))/(pow(X[3],n1)+(pow(KG1,n1)));
    w[1]=alphagal0G80 + alphagalG80*(pow(X[3],n80)/(pow(X[3],n80)+pow(KG80,n80)));
    w[2]=alphaG30 + alphaG3*(pow(X[3],n3)/(pow(X[3],n3)+(pow(KG3,n3))));
    w[3]=alphaG4;
    // Binding of Gal1 and Gal3 with galactose
    w[4]=kf1*X[0]*gal;
    w[5]=kf3*X[2]*gal;
    // Unbinding of Gal1-galactose and Gal3-galactose
```

```
w[6]=kr1*X[4];
w[7]=kr3*X[5];
// Binding of Gal1,3,4 with Gal80
w[8]=kf81*X[4]*X[1];
w[9]=kf83*X[5]*X[1];
w[10]=kf84*X[3]*X[1];
// Unbinding of Gal1-80,Gal3-80,Gal4-80
w[11]=kr81*X[6];
w[12]=kr83*X[7];
w[13]=kr84*X[8];
// Decay of all protein
w[14]=gamma*X[0];
w[15]=gamma*X[1];
w[16]=gamma*X[2];
w[17]=gamma*X[3];
w[18]=gamma*X[4];
w[19]=gamma*X[5];
w[20]=gamma*X[6];
w[21]=gamma*X[7];
w[22]=gamma*X[8];

// Calculate sum of reaction probability
int sizew=sizeof(w)/sizeof(w[0]);
double W=sumn(w,sizew);

// Stochiomentric matrix - Should equal to no of events
int S[23][9]= {{1,0,0,0,0,0,0,0,0},
{0,1,0,0,0,0,0,0,0},
{0,0,1,0,0,0,0,0,0},
{0,0,0,1,0,0,0,0,0},
{-1,0,0,0,1,0,0,0,0},
{0,0,-1,0,0,1,0,0,0},
{1,0,0,0,-1,0,0,0,0},
{0,0,1,0,0,-1,0,0,0},
{0,-1,0,0,-1,0,1,0,0},
{0,-1,0,0,0,-1,0,1,0},
{0,-1,0,-1,0,0,0,0,1},
{0,1,0,0,1,0,-1,0,0},
{0,1,0,0,0,1,0,-1,0},
{0,1,0,1,0,0,0,0,-1},
{-1,0,0,0,0,0,0,0,0},
{0,-1,0,0,0,0,0,0,0},
{0,0,-1,0,0,0,0,0,0},
{0,0,0,-1,0,0,0,0,0},
{0,0,0,0,-1,0,0,0,0},
{0,0,0,0,0,-1,0,0,0},
{0,0,0,0,0,0,-1,0,0},
{0,0,0,0,0,0,0,-1,0},
{0,0,0,0,0,0,0,0,-1},
};


// Start time loop from t=0 and end at t=10

double time=0;
double tau=0;
int count=0;
// float t[100000]={0};
// t[step]=time;

// Trajectory for each experiment
while (time<maxt)
{
    count=count+1;
    // Step2:
    // Generate random number bw 0-1
    float r1=(float) rand()/RAND_MAX;
    // printf("\n Random number 1: %f",r1);
```

```
        float r2=(float) rand()/RAND_MAX;
        // printf("\n Random number 2: %f",r2);
        // Calculate the next time interval
        tau=log(1/r1)/W;
        if(r1==0){
            //printf("\n tau is infi");
            float r1=(float) rand()/RAND_MAX;
            tau=log(1/r1)/W;
        }
        // Calculate next reaction
        int mu=0;
        float s=w[mu];
        float r0=r2*W;
        while (s < r0){
            mu = mu + 1;
            s = s + w[mu];
        }

        // Step3:
        // Update time and concentration
        int sizeX=sizeof(X)/sizeof(X[0]);
        for (int i=0;i<sizeX;i++){
            X[i]=S[mu][i]+X[i];
        }

        // Update time
        time=time+tau;
        // t[step+1]=time;
    // Update Propensity function for next calculation
    // Gal protein production (basal + induced)
    w[0]=alphaG10 + alphaG1*(pow(X[3],n1))/(pow(X[3],n1)+(pow(KG1,n1)));
    w[1]=alphagal0G80 + alphagalG80*(pow(X[3],n80)/(pow(X[3],n80)+pow(KG80,n80)));
    w[2]=alphaG30 + alphaG3*(pow(X[3],n3)/(pow(X[3],n3)+(pow(KG3,n3))));
    w[3]=alphaG4;
    // Binding of Gal1 and Gal3 with galactose
    w[4]=kf1*X[0]*gal;
    w[5]=kf3*X[2]*gal;
    // Unbinding of Gal1-galactose and Gal3-galactose
    w[6]=kr1*X[4];
    w[7]=kr3*X[5];
    // Binding of Gal1,3,4 with Gal80
    w[8]=kf81*X[4]*X[1];
    w[9]=kf83*X[5]*X[1];
    w[10]=kf84*X[3]*X[1];
    // Unbinding of Gal1-80,Gal3-80,Gal4-80
    w[11]=kr81*X[6];
    w[12]=kr83*X[7];
    w[13]=kr84*X[8];
    // Decay of all protein
    w[14]=gamma*X[0];
    w[15]=gamma*X[1];
    w[16]=gamma*X[2];
    w[17]=gamma*X[3];
    w[18]=gamma*X[4];
    w[19]=gamma*X[5];
    w[20]=gamma*X[6];
    w[21]=gamma*X[7];
    w[22]=gamma*X[8];
// Calculate sum of reaction probability
W=sumn(w,sizew);



    }

    fprintf(SS,"%lf \t %d \t %d \t %d \t %d \t %d \t %d \t %d \t %d \t %d\n",time,X
[0],X[1],X[2],X[3],X[4],X[5],X[6],X[7],X[8]);
    }
```

```
    fclose(SS);
}
    clock_t end = clock();
        time_spent += (double)(end - begin) / CLOCKS_PER_SEC;
        printf("\n---------------------------------------");
        printf("\nTime elpased is %f min", time_spent/60);
        printf("\n---------------------------------------\n");
    return 0;

}
```
